# Wnt5a, TLR2 and TLR4 are elevated in advanced human atherosclerotic lesions

**DOI:** 10.1007/s00011-013-0697-x

**Published:** 2013-12-18

**Authors:** Ramiro Malgor, Pooja M. Bhatt, Beth A. Connolly, Denise L. Jacoby, Kyle J. Feldmann, Mitchell J. Silver, Masato Nakazawa, Kelly D. McCall, Douglas J. Goetz

**Affiliations:** 1 Department of Biomedical Sciences, 202b Academic and Research Center, Heritage College of Osteopathic Medicine, Ohio University, Athens, OH 45701 USA; 2 Department of Biological Sciences, Molecular and Cellular Biology Graduate Program, Ohio University, Athens, USA; 3 The Diabetes Institute, Heritage College of Osteopathic Medicine, Ohio University, Athens, USA; 4 Department of Internal Medicine, Riverside Methodist Hospital, Columbus, OH USA; 5 Mid West Cardiology Research Foundation, Columbus, OH USA; 6 Office of Research and Grants, Ohio University, Athens, USA; 7 Department of Specialty Medicine, Heritage College of Osteopathic Medicine, Ohio University, Athens, USA; 8 Department of Chemical and Biomolecular Engineering, Biomedical Engineering Program, Ohio University, Athens, USA

**Keywords:** Inflammation, Atherosclerosis, Wnt5a, TLR4, TLR2

## Abstract

**Objective and design:**

Atherosclerosis (ATH) is a chronic inflammatory disease that involves cascades of signaling events mediated by various effector proteins. Here we sought to determine if the expression of Wnt5a, a secreted glycoprotein, is altered in discrete regions of the arterial plaque.

**Methods:**

Atherosclerotic plaque tissues from 14 human subjects undergoing elective carotid endarterectomy were used in this study. Immunohistochemistry and laser capture microdissection combined with quantitative real-time PCR were used to determine the expression of Wnt5a and Toll-like receptors (TLRs) in different sections of the arterial lesions. Atherosclerotic serum samples (*n* = 30) and serum from healthy subjects (*n* = 16) were quantified for Wnt5a using an enzyme-linked immunosorbent assay (ELISA).

**Results:**

The data analysis revealed that Wnt5a transcripts and protein were elevated in advanced arterial lesions relative to less advanced arterial lesions; that Wnt5a expression correlated with the presence of TLR4 and TLR2 transcripts; and that the average amount of Wnt5a protein present in atherosclerotic patient serum was significantly higher compared to healthy controls.

**Conclusions:**

This study is the first to provide evidence that the expression of Wnt5a increases as the disease progresses to a more advanced stage, and that this expression is coincident with that of TLR2 and TLR4. In addition, we found that the average Wnt5a levels in the serum of atherosclerotic patients are elevated relative to healthy controls, which is consistent with the hypothesis that Wnt5a plays a role in ATH.

## Introduction

Atherosclerosis (ATH) is a highly prevalent chronic systemic inflammatory disease [1]. A hallmark of ATH is the accumulation of distinct cell types (e.g. macrophages, smooth muscle cells and foam cells) in the arterial wall, along with a buildup of lipids, cholesterol, calcium, and cellular debris within the intima of large-sized and medium-sized muscular arteries. This process leads to the formation of fatty streaks, atheromas, or atherosclerotic plaques that have a potential to rupture, causing complications such as a stroke, myocardial infarction or peripheral vascular ischemia [2–4].

Monocytes/macrophages are the predominant types of immune cells in atherosclerotic plaques, and are known to significantly modify their local environment. Specifically, activated macrophages secrete different types of effector proteins (e.g., cytokines such as tumor necrosis factor-α, interleukin-12 and -18, chemokines like CCL3, CCL4) that can act on the macrophages themselves or on other local cell types (e.g. smooth muscle cells). The secretion of an initial set of proteins and their autocrine/paracrine effects can trigger a second wave of effector molecules that may favor the development of a severe plaque [5]. In this manner, cascades of signaling events transpire during ATH, mediated to a large extent by these potent effector proteins. Determining which effector proteins are present, and their location within a plaque, gives insights into the mechanisms of ATH and may suggest novel approaches to diagnosing and staging atherosclerotic plaques.

Wnts are a family of highly conserved, secreted glycoproteins that are involved in many biological processes such as cell fate, polarity and differentiation [6]. Over the last decade, Wnts have also been implicated in a variety of clinical conditions, including pathological inflammation [7–9]. Our research specifically focuses on Wnt5a, which has been implicated in rheumatoid arthritis [10], sepsis [11], and tuberculosis [7]. Wnt5a activity in pathological inflammation has been linked to Toll-like receptors (TLRs), a family of transmembrane signaling proteins that trigger the innate immune response [7, 12]. In particular, TLR4 and TLR2 have been implicated in the pathogenesis of ATH [13–16]. Recently, our group demonstrated that Wnt5a is specifically expressed in the macrophage-rich regions of murine and human atherosclerotic lesions, and that this expression was coincident with that of TLR4 [17]. We also demonstrated that Wnt5a transcripts are induced in murine macrophages upon stimulation with lipopolysaccharide (a trigger for TLR4 signaling) [17], and that oxidized-LDL stimulation of human monocytes/macrophages leads to increased expression of Wnt5a transcripts, indicating that macrophages are a source of Wnt5a in ATH [18].

Combined, the above observations led us to the hypothesis that Wnt5a is important in the pathogenesis of ATH, and that the expression of Wnt5a is linked to TLR. In order to probe our hypothesis more extensively and rigorously, we employed laser capture microdissection (LCM) combined with quantitative real-time PCR (RT-PCR), in addition to immunohistochemical analysis. By using LCM and quantitative RT-PCR, an additional and rigorous test of coincident expression of TLR4 and TLR2 with Wnt5a mRNA can be conducted, since the expression of each of these transcripts in discrete regions can be compared. We found that the expression of Wnt5a increases as the disease progresses to a more advanced state, and this expression is coincident with that of TLR2 and TLR4. Finally, we speculated that Wnt5a protein might be elevated in serum isolated from atherosclerotic patients. Thus, we compared the serum levels of Wnt5a in patients undergoing elective carotid endarterectomy to serum isolated from healthy controls, and found that the levels of Wnt5a were, on average, higher in the atherosclerotic patient samples.

## Methods

### Source and handling of human atherosclerotic tissue and serum prior to analysis

Atherosclerotic plaque tissue and peripheral blood were obtained from 14 and 30 human subjects, respectively, undergoing elective carotid endarterectomy at Riverside Methodist Hospital, Columbus, OH from 2009 to 2011. All samples were obtained and used in compliance with the approval of the Institutional Review Board for Human Subjects Committee at Ohio University and Riverside Methodist Hospital. Based on gross examination, each fresh tissue sample was divided into fragments that contained well-developed plaques and fragments where the wall of the artery looked less affected or even normal; both tissue fragments were processed separately. The samples were immediately fixed with 10 % buffered formalin overnight. Subsequently, samples were dehydrated in sequential alcohol/xylene washes and embedded in paraffin. Serum separation was performed from fresh whole blood, and collected serum was stored at −80 °C until further use. Clinical data are summarized in Table 1.

**Table 1:** Clinical and pathological characteristics

Total patients	*n* = 30
Age range	43–90 years
Average	68.2 (SD 11.3)
Gender M/F	13/17
BMI range	21.6–35.6
Average	28.3 (SD 4.4)
Tobacco	13/30
Cancer	5/30
Diabetes	10/30
Other inflammatory disease	15/30
Statin treatment	22/30

### Hematoxylin and eosin (H&E) staining

Tissue blocks were sectioned into 5 μm sections and analyzed using hematoxylin & eosin (H&E) staining. The microanatomy of the tissue section revealed by the H&E staining was used to classify the tissue according to the American Heart Association guidelines [19, 20]. Briefly, tissues with smaller lipid cores, a low number of foam cells, limited or no calcification and no complications (e.g. no fissures) were classified as less advanced. In contrast, plaques with a larger lipid core, substantial amount of inflammation (e.g. foam cells, lymphocytes, and/or giant macrophages), calcification, and other complications were graded as more advanced lesions. In an effort to control for variations in the tissue, the three analytical approaches used in this study, i.e. H&E, RT-PCR, and immunohistochemistry were conducted on tissue sections sequential to one another.

### Laser capture microdissection (LCM) and RNA extraction

Prior to slide preparation, all the equipment was cleaned to minimize any potential RNase contamination. To isolate total RNA from the microdissected tissues, paraffin blocks were sectioned into 8 μm sections using a rotary microtome. The first three sections were discarded and subsequent sections were removed from the microtome and put in a water bath with RNase free water. Afterwards, each section was mounted on a polyethylene naphthalene membrane slide (Leica, Wetzlar, Germany). Subsequently, the sections were deparaffinized in xylenes and rehydrated in graded alcohols (100, 90, and 70 % ethanol). The sections were then stained using H&E, and dissected using Leica LMD6000 (Wetzlar, Germany) according to the manufacturer’s instructions. The areas of interest for microdissection and RNA extraction were: region 1—less advanced region of the arterial wall; region 2—shoulder of the lesion usually containing a high number of foam cells; region 3—fibrous cap; and region 4—plaque/intima. RNA from the dissected tissue was extracted using Qiagen miRNeasy FFPE Kit according to the manufacturer’s instructions (Qiagen, Valencia, CA, USA). RNA was quantified, and reverse transcribed using a High Capacity cDNA Reverse Transcription Kit (Applied Biosystems, Foster City, CA, USA).

### Quantitative RT-PCR

Human HPRT1, Wnt5a, TLR4 and TLR2 transcripts were quantified using ABI Step One Plus quantitative RT-PCR (Applied Biosystems) for the LCM samples. Taqman Gene Expression Assays GEx (Hs99999909_m1 HPRT1, Hs00180103_m1 Wnt5a, Hs00152939_m1 TLR4, and Hs00610101_m1 TLR2) and Taqman Gene Expression Master Mix (Applied Biosystems) were used according to the manufacturer’s protocol. The thermal-cycler conditions were: 1 cycle of 95 °C for 10 min, 40 cycles of 95 °C for 15 s, 60 °C for 1 min. Comparative Delta Ct method was used to obtain fold expression of Wnt5a, TLR4 and TLR2 relative to HPRT1 endogenous control.

### Immunohistochemistry

Consecutive 5 μm sections from each block were placed on a single slide for immunostaining. One section served as an isotype-matched control for the other section on the same slide. Immediately prior to staining, sections were placed in an incubator at 60 °C for 1 h, and deparaffinized/rehydrated in xylenes/graded alcohol. After antigen retrieval in 10 mM citrate buffer pH 6.0, the sections were blocked with 3 % hydrogen peroxide followed by 1 % bovine serum albumin (Sigma Aldrich, St. Louis, MO, USA). Wnt5a was detected with monoclonal mouse anti-human Wnt5a (Abcam, Cambridge, MA, USA), while the isotype-matched control tissues were stained with normal mouse IgG (Santa Cruz Biotechnology Inc., Santa Cruz, CA, USA). These tissues were then incubated with HRP conjugated secondary sheep anti-mouse IgG (Amersham Biosciences, UK). DAB enhanced liquid substrate (Sigma Aldrich) was added for visualization. Subsequently, the slides were counter-stained with hematoxylin.

### Wnt5a enzyme-linked immunosorbent assay (ELISA)

Atherosclerotic serum samples were obtained from 30 human subjects undergoing elective carotid endarterectomy at Riverside Methodist Hospital, Columbus, OH, USA. Normal control serum (*n* = 16) was either obtained from healthy volunteers with informed consent, or purchased from Bioserve (Beltsville, MD, USA). All samples were obtained and used in compliance with the Institutional Review Board for Human Subjects Committee at Ohio University and Riverside Methodist Hospital. Commercially available Wnt5a ELISA kit (USCN Life Science Inc., Burlington, NC) was used to detect the expression of Wnt5a protein in the serum samples. The ELISA was performed according to the manufacturer’s protocol.

### Statistical analyses

Comparisons between different areas in the arterial wall were conducted using a Nonparametric test (Wilcoxon test), and the significance level was set to 0.05. Data for Wnt5a protein from a single serum sample is expressed as mean of duplicate samples. An unpaired student’s *t*-test was performed for comparison between the control and atherosclerotic serum samples. Correlation between Wnt5a serum levels, as measured by ELISA and clinical donor characteristics, was assessed using linear regression for age and body mass index (BMI), and Welch’s modified *t*-tests for gender, diabetes, cancer, inflammatory disease, and statin treatment. All tests were performed two-tailed with the typical 5 % significance level. Data were expressed as mean ± SD.

## Results

### Wnt5a mRNA transcripts are elevated in diseased regions of atherosclerotic arteries relative to the less advanced regions of the arteries

**Fig. 1:**
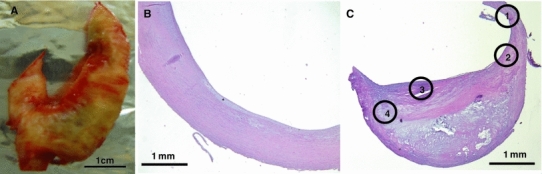
Example of the carotid artery samples included in this study. **a** Gross appearance of the carotid artery removed by endarterectomy before fixation; *Bar* 1 cm. **b** Microscopic view of the H&E staining of a cross section of the artery at the areas defined as less advanced; *Bar* 1 mm. **c** Microscopic view of the H&E staining of the cross-section of the artery at the areas defined as plaque or advanced; *Bar* 1 mm. Regions 1 through 4 show the sections analyzed by LCM/RT-PCR: region 1—less advanced region of the arterial wall; region 2—shoulder of the lesion usually containing a high number of foam cells; region 3—fibrous cap; and region 4—plaque/intima

Carotid artery segments, removed during elective endarterectomy, were obtained. A typical segment is shown in Fig. 1a, which reveals extensive plaque within the artery. These segments of the artery were formalin fixed, paraffin embedded and sectioned. A typical H&E staining of a less advanced arterial tissue and of an atherosclerotic plaque is shown in Fig. 1b and c, respectively. Figure 1c presents an axial view of roughly 50 % of the artery. The large white space subtended by the tissue is the lumen of the artery. The tissue contains four distinct regions: region 1—less advanced; region 2—shoulder of the lesion usually containing a high number of foam cells; region 3—fibrous cap; and region 4—plaque/intima.

**Fig. 2:**
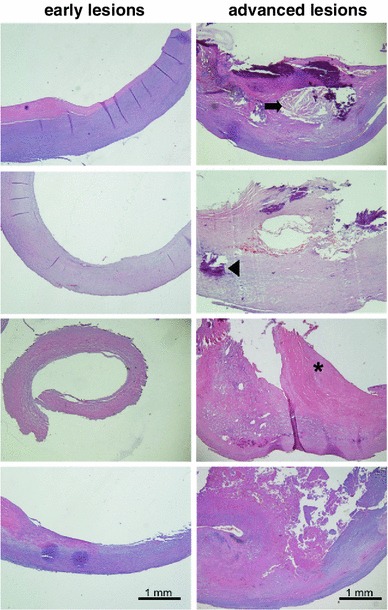
Examples of H&E staining of carotid artery samples from patients included in this study. The *left panel* in the figure is a cross section of the arterial wall from areas defined as less advanced lesions, whereas the *right panel* demonstrates a cross section of the arterial wall from areas defined as advanced lesions; *Bar* 1 mm. The *arrow* indicates a necrotic center; an *inverted triangle* indicates the areas with calcification; a *star* indicates the fibrous cap region

Figure 2 shows representative sections of human carotid arterial wall from four different patients used in this study. Areas of the artery free of plaque or with less evidence of disease reveal a thin intima with a preserved architecture of the arterial wall, and were classified as less advanced. Areas of the artery defined as advanced lesions demonstrated a fibrous cap, necrotic center, inflammatory cells and calcifications.

**Fig. 3:**
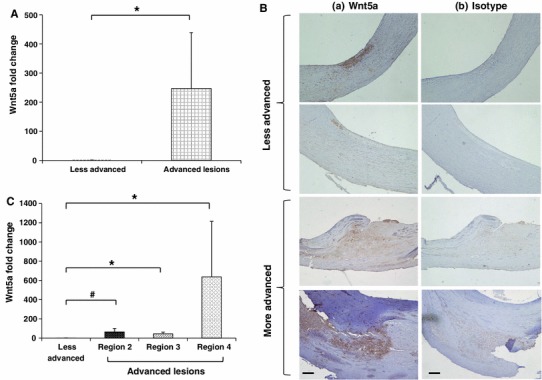
Quantitative RT-PCR and immunohistochemistry revealed higher expression of Wnt5a in diseased regions of the atherosclerotic arteries relative to less advanced regions. LCM was used to obtain tissue Sections 1–4 described in the “Materials and methods” section. RNA extracted from these samples was reverse transcribed and subjected to quantitative RT-PCR. **a** Mean Wnt5a fold change for 14 different human subjects relative to HPRT1 endogenous control. **b** Tissue sections generated from the carotid atherosclerotic lesions (*n* = 14) were treated with anti-Wnt5a (*a*) or isotype control (*b*), and analyzed by immunohistochemistry for the presence of Wnt5a; *Bar* 200 μm. **c** Individual comparison of 14 samples for Wnt5a transcripts in less advanced regions of the arterial tissue (region 1) compared to the three different advanced regions, i.e. regions 2, 3 and 4. Results are expressed as mean ± SEM. *P < 0.002, ^#^ *P* < 0.005 compared to less advanced regions of the arteries, as determined by nonparametric Wilcoxon test

We have previously demonstrated that Wn5a protein is present in human atherosclerotic tissue [17]. We used the above described tissue sections to expand this observation. Specifically, we utilized LCM, in conjunction with quantitative RT-PCR, to determine if Wnt5a transcripts are elevated in regions of the arteries that clearly have plaque present compared to less advanced regions of the arteries. Samples were taken, via LCM, from regions 2, 3 and 4, (depicted in Fig. 1c), and the level of Wnt5a mRNA determined via quantitative RT-PCR. The average of these values was compared to that obtained using samples taken from the less advanced regions of the arteries (region 1 in Fig. 1c). As shown in Fig. 3a, the average expression (*n* = 14) of Wnt5a transcripts is significantly and dramatically higher in regions of the artery that are diseased, relative to the less advanced regions of the artery (*P* < 0.002). This finding at the mRNA transcript level was paralleled at the protein level. Specifically, immunohistochemistry revealed significant staining for Wnt5a in diseased regions of the artery compared to less advanced regions. As expected, the isotype matched control stained negative for Wnt5a (Fig. 3b).

We next sought to determine if the expression of Wnt5a varied between the diseased regions, i.e. between regions 2, 3, and 4 depicted in Fig. 1c, of the plaque. As shown in Fig. 3c, the average expression (*n* = 14) of Wnt5a transcripts was significantly elevated in each of the diseased regions relative to the less advanced regions. Thus, significant Wnt5a transcript levels were observed in the plaque/intima, the fibrous cap (*P* < 0.002), and the shoulder of the lesion (*P* < 0.005). Note that the plaque/intima region appeared to have the highest level of Wnt5a transcripts. That said, there was also a large amount of variability in the results from this region, and a statistical analysis comparing this region to the other diseased regions revealed no significant differences. The high variability could be due to the presence of a diverse population of cell types (e.g. lymphocytes, smooth muscle cells).

### TLR4 and TLR2 mRNA transcripts are elevated in diseased regions of the arteries relative to less advanced regions of the arteries

**Fig. 4:**
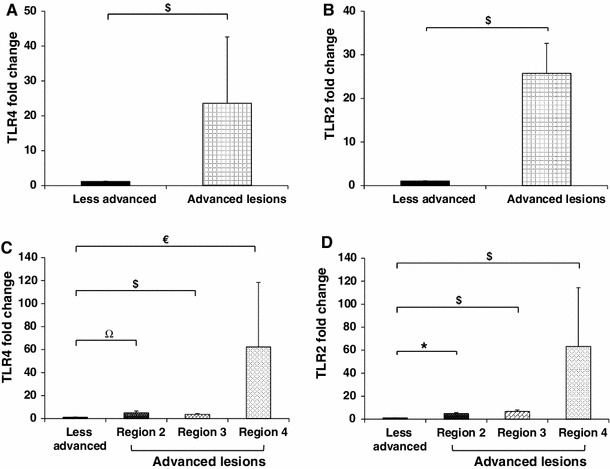
Quantitative RT-PCR revealed higher expression of TLR4 and TLR2 in diseased regions of the atherosclerotic arteries relative to less advanced regions. RNA isolated (*n* = 14) from the areas defined in Fig. 1 was reverse transcribed, and subjected to quantitative RT-PCR for analyzing the transcription levels of TLR4 **(a)** and TLR2 **(b)** in all four regions using HPRT1 as an endogenous control. Individual comparison of 14 samples for TLR4 **(c)** and TLR2 **(d)** transcripts in less advanced arterial tissue (region 1) compared to the three different advanced regions, i.e. regions 2, 3 and 4, was also performed. Results are expressed as mean ± SEM. ^$^ *P* < 0.001, *P < 0.002, ^€^ *P* < 0.003, ^Ω^ *P* < 0.004 compared to less advanced regions of the arteries, as determined by nonparametric Wilcoxon test

We have previously reported coincident TLR4 and Wn5a protein expression in human atherosclerotic tissue [17]. We sought to further investigate this observation by assaying for TLR4 mRNA transcript expression in the carotid artery samples using LCM and quantitative RT-PCR. We also assayed for TLR2 in this analysis, since TLR2 has also been implicated in ATH [14, 21, 22]. As shown in Fig. 4a and b, respectively, the average expression (*n* = 14) of TLR4 and TLR2 transcripts are significantly higher in regions of the arteries that are diseased, relative to less advanced regions within the same artery (*P* < 0.001).

We next sought to determine if the expression of TLR4 and TLR2 varied between the diseased regions, i.e. between regions 2, 3, and 4 depicted in Fig. 1c of the plaque. As shown in Fig. 4c and d, respectively, the average expression (*n* = 14) of TLR4 and TLR2 were significantly elevated in each of the diseased regions relative to the less advanced regions. Thus, significant TLR4 and TLR2 transcript levels were observed in the plaque/intima, the fibrous cap, and the shoulder of the lesion (*P* ≤ 0.004). As we observed with Wnt5a (Fig. 3c), the plaque/intima region appeared to have the highest level of TLR4 and TLR2 transcripts. That said, there was also a large amount of variability in the results from this region, and a statistical analysis comparing this region to the other diseased regions revealed no significant differences. We suspect that the source of the variability in the plaque/intima region is due to extensive cellular/molecular heterogeneity within this region of the plaque.

### Wnt5a protein in serum isolated from patients with symptomatic ATH is higher than in serum isolated from normal subjects

**Fig. 5:**
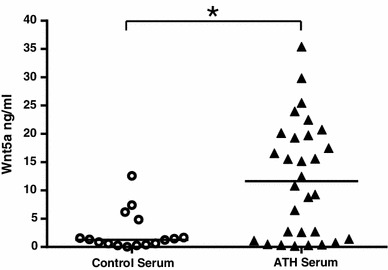
Atherosclerotic patients show increased levels of Wnt5a protein in their serum. ELISA was used to quantify the level of Wnt5a protein in serum samples from normal (*n* = 16) and atherosclerotic subjects (*n* = 30). The results are expressed as mean concentration values (ng/ml) of the duplicate samples. *P ≤ 0.0005 compared to control serum as determined by unpaired student’s *t*-test

The above observations support our hypothesis that Wnt5a is present and elevated at sites of ATH and plays a role in the disease process. Given the increased expression of Wnt5a, and the fact that Wnt5a is a secreted protein and present in the vessel wall and thus proximal to the blood stream, we questioned whether Wnt5a protein might be elevated in patients suffering from symptomatic ATH. Thus, we isolated serum from 30 atherosclerotic patients (14 of whom belonged to the same cohort of patients from which the tissue samples were drawn) and 16 healthy volunteers. We then quantified the level of Wnt5a protein using a commercially available Wnt5a ELISA. The average Wnt5a protein expression was significantly higher (*P* ≤ 0.0005) in serum samples from patients with atherosclerotic disease compared to serum samples from healthy controls (Fig. 5). Interestingly, there appeared to be two populations of atherosclerotic patients in regards to Wnt5a expression. Approximately half the population had Wnt5a protein levels similar to the healthy controls and the other half had Wnt5a protein levels that were higher than those observed in healthy controls (Fig. 5). We thus investigated the presence, or lack thereof, of an effect of clinical characteristics and elevated Wnt5a observed in the serum of atherosclerotic patients. The clinical/pathological characteristics of the population of patients are summarized in Table 1. Interestingly, we found that only a previous history of cancer had a statistically significant (*P* ≤ 0.01) effect on the level of Wnt5a present in the serum. Note that five patients had a previous history of cancer. When Wnt5a results from these five patients were removed from the patient population, there was still a statistically significant difference between the Wnt5a levels observed in the healthy controls relative to those observed in the atherosclerotic patients (*P* ≤ 0.002). This final analysis demonstrates that the difference between the atherosclerotic patient population and the healthy controls was not due to the presence of cancer in patients in the atherosclerotic population.

## Discussion

This study demonstrates that the expression of Wnt5a transcripts is higher in areas of the arterial wall that show severe histopathological alterations corresponding to advanced human carotid atherosclerotic lesions. This observation is congruous with the immunohistochemical data, which revealed increased expression of Wnt5a in the regions of severely diseased carotid arteries as compared to less advanced regions within the same artery. In addition, using LCM together with quantitative RT-PCR, we found significantly increased expression of Wnt5a transcripts in region 2—shoulder of the lesion usually containing a high number of foam cells; region 3—fibrous cap; and region 4—plaque/intima. These findings confirm and extend our previous study, where we observed increased expression of Wnt5a protein in murine and human atherosclerotic lesions mainly consisting of macrophages/foam cells [17].

TLRs, specifically TLR4 and TLR2, have been implicated in ATH, and can be induced by endogenous (e.g. activated inflammatory cells) or exogenous (e.g. bacteria) ligands. Both TLR4 and TLR2 have been shown to be highly expressed in atherosclerotic lesions [13, 17, 22], and are thought to play a role in atherosclerotic inflammation, matrix degradation, arterial remodeling, and plaque instability and rupture [16, 22, 23]. We previously reported co-localization of TLR4 with Wnt5a at the protein level [17], and here we report the elevated expression of TLR4 and TLR2 mRNA in the diseased regions of the arteries relative to less advanced regions. In order to determine whether the expression of TLR4 and TLR2 varied between different regions within a plaque, LCM in conjunction with quantitative RT-PCR was used. We found increased expression of both of these receptors in region 4—plaque/intima region, although they were also expressed in regions 2 and 3 at a relatively lower concentration.

While increased Wnt5a expression has been reported in atherosclerotic lesions, the exact role of Wnt5a in this disease process is still unknown and is an interesting topic of investigation [17, 18, 24]. Recently, a possible role of Wnt5a in vascular calcification has been reported [25]. Other studies have shown that Wnt5a is upregulated in certain inflammatory diseases (e.g. rheumatoid arthritis, tuberculosis, and sepsis), and particularly by activated macrophages [7, 10, 11, 18, 26]. Additionally, a link between TLR/Wnt5a signaling pathway activation has been proposed as a possible mechanism involved in the pathogenesis of these inflammatory disorders [16, 27–29]. Blumenthal et al. [7] reported that TLR2 and TLR4 activated by mycobacteria and lipopolysaccharide, respectively, led to the upregulation of Wnt5a mRNA expression in human macrophages. Interestingly, the antagonists for both TLR2 and TLR4 were shown to inhibit bacteria-induced upregulation of Wnt5a mRNA [7]. Based on these reports, it seemed plausible to explore the hypothesis that TLR2, TLR4, and Wnt5a may participate together in the pathogenesis of ATH. Indeed, our results demonstrate relatively higher levels of mRNA for Wnt5a, TLR2, and TLR4 in the diseased regions compared to the less advanced regions of the artery. Although additional work is required to ascertain the combined role of Wnt5a, TLR2 and TLR4 signaling in ATH, our finding of coincident expression suggests a synergistic role of these mediators in disease pathogenesis.

Since Wnt5a is a secreted protein, we sought to determine if Wnt5a could be detected in the serum of human atherosclerotic patients. We found increased levels of Wnt5a protein in human atherosclerotic serum samples as compared to control serum samples, suggesting an active role of Wnt5a in the pathogenesis of ATH. Interestingly, approximately half the atherosclerotic population had Wnt5a protein levels similar to the healthy controls, and the other half had Wnt5a protein levels higher than those observed in healthy controls. One plausible interpretation of the “bimodal” data is that Wnt5a serum levels identify subpopulations of atherosclerotic patients. A further investigation into the relationship between Wnt5a in serum and vascular disease, and its possible clinical implications, is clearly required and warranted.

In considering the Wnt5a serum results, it is important to recognize that Wnt5a has been reported to be increased in other disease settings. For example, Bilkovski et al. [30] found elevated Wnt5a protein in adipose tissue from obese and type II diabetic patients, as well as elevated Wnt5a mRNA in circulating CD14^+^ monocytes isolated from these patients. The patient population in the present study contained diabetic patients, as well as patients with other inflammatory diseases (Table 1). Although we did not find a correlation between Wnt5a levels and the other diseases present in the ATH patient population used in this study, except, as noted, for cancer, the presence of these diseases does confound our interpretation. Based on the data set we currently have, we cannot pinpoint the exact clinical cause for the elevated Wnt5a. That said, our finding that the Wnt5a levels are statistically higher in atherosclerotic patients compared to controls is consistent with the hypothesis that Wnt5a plays a role in ATH.

In conclusion, these results confirm and extend our previous report that Wnt5a is expressed in atherosclerotic lesions. This study is the first to provide evidence that the expression of Wnt5a increases as the disease progresses to a more advanced stage, and that the expression of Wnt5a, TLR2, and TLR4 is coincident in the diseased regions. In addition, we found that the average Wnt5a levels in the serum of atherosclerotic patients is elevated relative to healthy controls, which is consistent with the hypothesis that Wnt5a plays a role in ATH.
